# Supplementary material for: Patient acceptability of circulating tumour DNA testing in endometrial cancer follow‐up
Source: Eur J Cancer Care (Engl). 2021 Feb 22;30(4):e13429. doi: 10.1111/ecc.13429 (PMC11475366; doi:10.1111/ecc.13429)
Supplement: Supplementary file 1 — Supplementary Material [file ECC-30-e13429-s001.docx]

**Final themes template analysis**

1. **Motivation for taking part in the study**
   1. Altruism/thinking about others
   2. Protecting others
   3. Dislike of the internal examinations
2. **Experiences of taking part in the study**
   1. How the consultant introduced the study to the patient
   2. Extent of understanding the purpose and function of the blood test
   3. Any concerns about taking part
   4. Did taking part in the study make you worry more about your cancer?
3. **The utility and acceptability of ctDNA**
   1. How participants find having a blood test
   2. Personal willingness to have a blood test to monitor cancer recurrence instead of a clinical examination
   3. Comparison of the blood test to a gynaecological examination
      1. Preference for the blood test
4. **Follow-up experiences and preferences**
   1. Experience of follow-up care
   2. Apprehension leading up to hospital appointments
   3. Downsides to regular hospital follow-up
   4. Positives to regular hospital follow-up
   5. Travel to the hospital
5. **PIFU as a potential follow-up tool**
   1. Thoughts about PIFU as a scheme
      1. Positive reactions
      2. Negative reactions
      3. Neutral reactions
   2. Barriers to PIFU
   3. Benefits to PIFU
   4. Views on ability to contact the specialist team
   5. Willingness to be transferred to PIFU (hypothetically)
   6. Willingness to have the blood test along with PIFU
   7. Feelings if blood test results were raised
      1. Blood test results raised but nothing on scans yet
      2. Would you rather know your levels were raised or not?
