# Supplementary material for: Patient acceptability of circulating tumour DNA testing in endometrial cancer follow‐up
Source: Eur J Cancer Care (Engl). 2021 Feb 22;30(4):e13429. doi: 10.1111/ecc.13429 (PMC11475366; doi:10.1111/ecc.13429)
Supplement: Supplementary file 2 — Supplementary Material [file ECC-30-e13429-s002.docx]

**Interview schedule/topic guide**

Introductions

Background to project and explanation of project aims

Complete consent form and answer any questions, ensure that participants have read and understand the participant information sheet

House keeping

**About you**

Please can you tell me a little bit about your medical condition and current stage of treatment/follow up

**Expectations of the EC blood test**

- Why did you agree to take part in the study?

*Promt – What was your main motivation?*

- To what extent did you understand what the blood test is able to detect?

-How did your consultant introduce the idea of the study to you?

*Prompt – How did this make you feel? Did you experience any concerns? What were these concerns? Why did you think in this way?*

- What has been your experience since taking part in the study?

- Has taking part in the study made you worry more about your cancer?

*If Yes –* what is it that is worrying? Is having a blood test worrying?

*If No – why do you think that is*?

**Follow up**

**-** What is your experience of follow up for endometrial cancer so far?

*Prompt – Do you find your appointments benefit your health?*

- Are there any downsides with regular hospital follow up?

*Prompt – How easy is it for you to travel to the hospital?*

*Prompt – Do you feel any anxiety about or in the lead up to hospital appointments?*

*-* Are there any positives with regular hospital follow up?

- What role does your GP play in your cancer follow up?

*Prompt – if you developed a new symptom that could be related to your cancer would you first see your GP or contact the hospital team directly?*

If the blood test is shown to accurately detect womb cancer would you be happy to have a blood test to monitor your cancer rather than a clinical examination?

*- Prompt – do you find a blood test more uncomfortable than a gynaecological examination?*

**Patient-initiated follow up**

- Have you heard of patient-initiated follow up (PIFU)?

*Prompt – this is where patients do not have routine follow up appointments but they have open access to the hospital specialist team if they develop a symptom or are concerned.*

- What are you thoughts about such a scheme?

- Do you think there are barriers/benefits to PIFU?

*Prompt – do you think there are people who might find it difficult to contact the specialist team if they had a problem?*

- Would you be happy to be transferred to a PIFU scheme?

*Prompt – what would be your main motivation for your decision?*

- If the blood test is shown to accurately detect womb cancer would you be happy to have a blood test to monitor your cancer and be transferred to PIFU rather than hospital follow up?

- The blood test we are developing shows the presence of cancer around six months before a patient may get symptoms or any signs detected by a scan. If you were having the blood test and the result was raised, how would that make you feel?

- You would be invited to attend the clinic for a scan. If an examination or CT scan didn’t show a sign of cancer, how would that make you feel?

- You would then have further FU appointments while waiting for the cancer to be detected – how would that make you feel? Would you rather know or not if your blood test results were raised?

Any questions?
